# The Immune System and Responses to Cancer: Coordinated Evolution

**DOI:** 10.12688/f1000research.6718.3

**Published:** 2021-01-27

**Authors:** Brendon J. Coventry, Maciej Henneberg

**Affiliations:** 1 Discipline of Surgery, Royal Adelaide Hospital, University of Adelaide, Adelaide, South Australia, 5000, Australia; 2 Biological Anthropology and Comparative Anatomy Unit, University of Adelaide, Adelaide, South Australia, 5005, Australia; 3 Institute of Evolutionary Medicine, The University of Zurich, 8057 Zurich, Switzerland

**Keywords:** Immune system, evolution, cancer, mutation, immune response, immunosurveillance, immunotherapy

## Abstract

This review explores the incessant evolutionary interaction and co-development between immune system evolution and somatic evolution, to put it into context with the short, over 60-year, detailed human study of this extraordinary protective system. Over millions of years, the evolutionary development of the immune system in most species has been continuously shaped by environmental interactions between microbes, and aberrant somatic cells, including malignant cells. Not only has evolution occurred in somatic cells to adapt to environmental pressures for survival purposes, but the immune system and its function has been successively shaped by those same evolving somatic cells and microorganisms through continuous adaptive symbiotic processes of progressive simultaneous immunological and somatic change to provide what we observe today. Indeed, the immune system as an environmental influence has also shaped somatic and microbial evolution. Although the immune system is tuned to primarily controlling microbiological challenges for combatting infection, it can also remove damaged and aberrant cells, including cancer cells to induce long-term cures. Our knowledge of how this occurs is just emerging. Here we consider the connections between immunity, infection and cancer, by searching back in time hundreds of millions of years to when multi-cellular organisms first began. We are gradually appreciating that the immune system has evolved into a truly brilliant and efficient protective mechanism, the importance of which we are just beginning to now comprehend. Understanding these aspects will likely lead to more effective cancer and other therapies.

## Introduction and Overview

It often goes unappreciated that the adaptive immune system developed hundreds of millions of years ago, and has evolved into a truly efficient protective mechanism, the importance of which we are just beginning to now understand in science and medicine. Acute immune responses have developed and evolved alongside infection and genetic diversity, as part of the entire evolutionary process of matching organism against organism. There has been a continuous development of the immune system's capacity to protect an organism against infections through rapid genetic somatic mutations that also led to a dynamic, intricate interplay between genetic endowment and somatic mutations. The immune system acts as an ultimate high fidelity 'read-out' for cellular genetic change, detecting cellular aberration at the molecular level in its very early stages as it develops, to remove or destroy aberrant cells. Such aberration arises from infection of cells by viruses, bacteria or other microbes, and from DNA damage, failed repair mechanisms, mutagens, carcinogens including UV light, toxins and other chemicals, and from cellular damage and ageing, thus embracing a wide variety of mechanisms causing cellular aberration. Aberration is detected by a variety of innate non-specific immune mechanisms through damage (or danger)-associated molecular patterns (DAMPs) that are released in response to trauma or injury to tissues, such as heat-shock proteins (HSPs) when cancer cells are injured; and also pathogen associated molecular patterns (PAMPs) which are a variety of molecules associated with (extrinsic) pathogens usually recognized by the innate immune system through pattern-recognition receptors (PRRs), such as toll-like receptors (TLRs) which include bacterial lipopolysaccharides, glycoconjugates, viral proteins and endotoxins. This might explain why addition of bacterial or viral antigens to cancer microenvironments (naturally or therapeutically) is observed to trigger successful adaptive immunity by supplying a suitable ‘danger signal’ in some situations. It is unclear whether the immune response is against the pathogen signal primarily, with secondary tumour killing; or drives forward augmenting a pre-existing immune response against the cancer antigens by releasing inhibition. The recent anti-PD-1 data would suggest the latter ^1^.

Many pathogens contain intrinsic danger signals, due to an evolutionary adaptation selected-for over millions of years, capable of inducing an immune response in animal and human immune systems. Constant dynamic interaction occurs between cells and the immune system to preserve homeostasis. Because the rate of mutation during cell division and tissue turnover far exceeds the rate of malignant tumour diagnosis, the immune system must serve in detecting and eliminating aberrant and frankly malignant cells at a developmentally early stage. MacFarlane Burnet proposed active immune surveillance occurs to detect cancer cells and virally infected cells to remove them from the body ^2– 7^. However, the fact that cancer does occur in humans and animals indicates that the process of detection and removal of malignant cells is not completely efficient. The reason for cancer persistence likely resides with observations that in the chronic state of antigen persistence the immune system continually appears to repeatedly ‘close itself down’ to avoid over-activation and to conserve energy. In the acute state, with exposure to each new pathogen the immune system responds rapidly over several days and then typically retains 'memory' of that encounter, enabling more rapid responses upon subsequent exposure. If the antigen can be acutely removed from the system, the immune system returns to its steady basal state via homeostatic mechanisms. However, if the antigen persists and cannot be removed from the organism, the immune system responds again and again with further cycles of activity. Over-reactivity is limited by eliciting an inhibitory response after each activation response, in the form of negative feedback for biological homeostatic damping. This cyclic feedback phenomenon is seen right across many, if not all, biological systems in nature. In the chronic state, the immune system repeatedly activates in response to persistent antigenic signals. When the antigenic signal cannot be removed with a second 'round' of activation, another cycle of activation and then inhibition occurs. This repetitive cycle continues until the antigenic focus is eventually removed, or the organism dies. Although this is an efficient system in the acute setting, in the chronic setting where the problem persists indefinitely and does not appear to resolve, 'chronic inflammation' can arise which is often far less energy efficient. Vast amounts of energy can be consumed in chronic severe inflammatory conditions ^1^. In areas of the world where infection has been effectively reduced by sanitation and other public health measures, non-infectious chronic inflammatory diseases have emerged as the major causes of morbidity and mortality. Clinically, this manifests as a relapsing and remitting process, often with malaise and weight loss characteristic of many chronic illnesses. The ‘immunological damping’, although on one hand offering some anti-inflammatory advantage, results in rather maladaptive processes which consume excessive amounts of energy, that may damage surrounding tissues and cells. The relapsing and remitting phenomenon is widely observable in states including chronic infections, chronic inflammatory diseases (like rheumatoid arthritis, thyroiditis and multiple sclerosis), and cancers. Over a number of generations, natural selection has led to efficiency improvements of immune responses. For instance, some specific chronic infections through co-adaptation of hosts and pathogens allowed normal (asymptomatic) functioning of most infected individuals; examples are endemic treponematoses ^8– 10^ or tuberculosis ^11, 12^.

This article considers the immune system more widely and relates cancer immunology in terms of the symbiotic evolutionary relationships between the immune system, somatic cell evolution, microbial pressure and chronic inflammation.

## Developmental Importance, Genome Diversity and Evolutionary Change

The immune system functions diversely across many organs to protect and maintain health. Importantly, the host’s immune system can regulate the genomic integrity across species and generations. Protection extends to all body barrier interfaces between the external and internal environment, where invasion of microbial agents is prevented or dealt with. The protection also acts against deleterious somatic mutations of host cells. The immune system is vital for maintenance of the health of all other body systems.

Essentially, the process of DNA-based evolution, besides adapting organisms to their physical environments, has pitted organism against organism in the quest for ultimate survival. According to Darwinian principles, the surviving organisms are the most successful in their reproduction either by conquering and terminating competing organisms, or in reaching symbiotic balance with them. That process requires protection of host DNA and also facilitates relatively rapid genomic constitutional adaptation by acquiring and modifying useful DNA from the environment ^13^. Indeed, the organism’s DNA is added to, modified and diversified to keep ahead of the 'genetic superior adaptability game' by mutation, plasmid transfer, viral transduction, mitotic translocations, and meiotic acquisition to allow its successful reproduction. The immune system undergoes constant modification of innate and adaptive immunity with exposure to antigenic stimuli both at the individual and the population levels.

The mammalian immune system represents one of the final (to date) central arbiters over the course of human Darwinian evolution. Many of the advances necessary for human adaptation have been moderated, directed and shaped by the influence of the immune system. Most fundamentally, the defense against infection and therefore survival of individuals to permit reproduction, is underpinned by immune system function. Less obvious, though equally fundamental, is the role of the immune system in maintenance of the organism’s homeostasis through removal of cells whose somatic mutations made them deleterious or disadvantageous. Natural selection applies not only to the successful reproduction of entire organisms, but also to the clonal reproduction of cell lineages, both cancer and immunological, within an organism ^14^.

The genes coding for the hypervariable regions of the antibody molecule and the genes for the variable regions of the T-cell receptor, mutate at a much faster rate than somatic genes under usual environmental pressure. The critical process of V(D)J gene recombination during T and B lymphocyte development randomly assembles different gene segments – named variable (V), diversity (D) and joining (J) genes, to generate immunoglobulin molecules and T cell receptors that can recognise antigens to which either the host or its genetic ancestors might have had exposure permitting binding and responsiveness to both new or changing antigens. These processes allow for more diverse binding patterns for antigens by both antibodies and T-cell receptors, which has significant immune adaptational advantage ^15, 16^. Somatic mutation on the other hand is a relatively slow process where genetic changes through (often) weaker selection pressures on survival and reproduction usually require some generations of cell divisions and progeny. These, changes, however, perceived as “millenial” can happen over just a few generations ^17– 19^. The immune system genes, however, constantly rapidly mutate in order to generate diverse conformations capable of binding the multitude of antigens to which an individual is exposed. Many of those antigens might be associated with threat and danger, for example, from microbial invasion. Examples of the somatic hypermutation process in B-cells are antibody class switching, and hypermutation of the genes for the variable regions of immunoglobulin molecules permitting the rapid antibody recognition of new foreign antigens with different molecular shapes. The immune system design has necessarily evolved, through continuous successive approximation, to detect subtle molecular cell surface aberrations. This occurs through both non-specific, and specific B- and T-cell, mechanisms in an elegantly integrated manner.

## How the Genome Monitors Itself and Evolves

Somatic changes of organisms generally occur at a gradual pace of varied rates as part of the slow, but effective, evolutionary process through such mechanisms as random mutation, natural selection and viral infection. For example, human morphological characteristics, like stature, brain size and tooth size change at rates ranging from 0.3 darwins to 65 darwins ^17^. Microbial DNA sequences, for example from retroviruses like HIV, Herpes viruses and *Mycobacterium tuberculosis*, have been identified in the human genome, and these genes must have been structurally incorporated over time from repeated exposure, interaction and exchange between mammalian and microbial DNA ^20, 21^. Human Endogenous Retroviruses (HERVs) are estimated to make up to 8% of the human genome, though fragmented and replication incompetent, it bears testament to long and intimate genetic interactions between a parasite with a few genes and 10,000 nucleotides, and a host of some 22,000 genes and some 2.85 billion nucleotides ^22– 24^. Interestingly, the (uninfected) C57 black mouse has several whole genomic copies of the LMP56 retrovirus in its germline ^23^. Clearly, the retrovirus became inserted into the murine genetic complement in the mammal's evolutionary past ^23– 27^. When infected with the virus in the experimental situation, the mouse develops a chronic immunodeficiency disease, the clinical course of which parallels HIV/AIDS in humans ^28^. It is now suggested that this chronic disease state is due to the murine immune system failing to differentiate between self and non-self, such that it homeostatically attenuates or down-regulates the immune response against the virus *in vivo* ^29, 30^. Failure to resolve the disease is due to persisting viral (self) antigens. The experimental similarity to the immune response in murine cancer models is strikingly compelling. In the case of cancer in the mouse the persisting antigens are due to the growing cancer which appears to exert a similar attenuating effect ^31^.

Over the millennia the constant exchange of genetic material between host and environmental microorganisms has offered incremental adaptive advantage to both organisms (host-pathogen adaptation), but in fundamentally different ways, perhaps comprising the ultimate symbiotic relationship, since both have evolved and survived ^32^. There remains a debate over whether the presence of microbial DNA is the result of incorporation or failed gene loss of inherited genes as evolution has occurred ^33– 36^. However, many microorganisms can expand rapidly, possess mechanisms for evasion of host defences, and can mutate at a rate that far outpaces somatic evolutionary change via much faster division/reproduction rates. This may explain the immune system’s evolved ability to match these rapid microbial mutational rates to more effectively neutralize them via innate mechanisms, antibody production and cellular responses. Examples are the microorganisms that rapidly expand and produce outbreaks of disease in humans, animals (from insects to mammals), and, plants, sometimes with transmission across species. Rapid, immediate 'revolutionary' adaptive change is advantageous to keep the immune system ahead of microbial mutation, virulence and growth ^37^. To oppose mutated, infected and otherwise aberrant cells, the immune system has a number of adaptive and protective mechanisms. These include somatic hypermutation genes for generation of hypervariable region binding domains for antibody molecules produced by plasma (B-) cells, and for the process of genetic recombination by T-cells forming the variable regions of T-cell surface receptors for rapid response to antigen exposure. In this way, adaptive immune responses can rapidly generate multiple molecules with variable affinity for binding whole or fragmented antigens. An analogy would be 'random number generation' to break undeciphered digital codes, or in contemporary terms to 'hack into' a computer system across encrypted firewalls ^38– 40^.

Without adequate host organism defence, infection would cause cellular damage and death. Humans are estimated to harbour some 10 ^14^ microbes, mostly bacteria, while we consist of only 10 ^13^ mammalian cells ^41– 43^. It might therefore be argued that in a cellular sense we are more bacterial than mammalian in constitution. The human body, like any other multicellular organism, should be treated as a complex ecosystem whose balance is dynamically maintained by feedback interactions amongst its parts.

To understand the human immune system, we must appreciate that each facet of the immune system has evolved concurrently as life itself has evolved. The mammalian genome, therefore constantly monitors itself through the actions of the immune system, both non-specific and adaptive. This is in order to achieve a state of evolving homeostasis to achieve progressive protection of the genome, and of cellular and tissue function, as the environmental, microbial and other pressures continually change.

## History of Immune System Development and Cellular Aberration

Life on earth commenced between 3 and 4 Ga (giga or billion years ago; 3–4×10 ^9^ years) as unicellular organisms (fungi, bacteria and viruses) adapted to survive environmental hazards through rapid reproduction, colony formation and repopulation. At some 1.2 Ga algal mats developed as the first multicellular organisms, and then about 1 Ga more complex chlorophyll-containing organisms evolved. About 450 Ma (mega or million years ago) even more complex plants developed and acquired fundamental innate static immune systems largely through intracellular anti-microbial molecules to resist infection principally from existing fungi, bacteria and viruses.

Recent studies indicate that sponges and other invertebrates developed both innate and adaptive immunity at around 800 Ma ago so that from that period marine sponges, coelenterates, gastropods (snails & slugs) and helminths (worms) possessed the ability for allograft rejection, indicating distinction between self and non-self ^44, 45^. A further advance in adaptive immunity developed with vertebrates around 450 Ma in primitive fish and amphibians, and with reptiles, about 300 Ma, this evolved rapidly for protection against infection.

Mammalian life began about 120 Ma, with immune system evolution to meet the need for local and systemic protection from invasive microorganisms, and with placentation ^32^. Indeed, for effective adaptive symbiosis the mammalian immune system must have developed evolutionary tolerance for specific microorganisms since some organisms conferred adaptive advantages and others did not. A part of these adaptations may have been inherited from therapsid ancestors.

**Figure 1.**
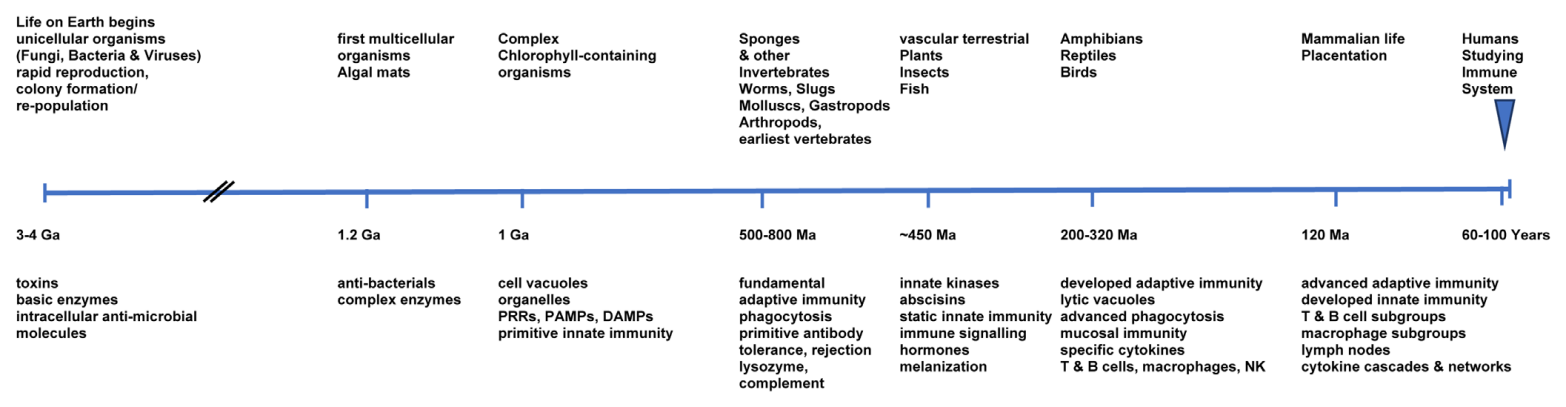
Timeline of estimated and known evolutionary developments of Life on Earth, and parallel changes in development of the immune system components [Ga = Giga/ billion / 10 ^9^ years; Ma = Mega/ million /10 ^6^ years; PRR = pattern-recognition receptors; PAMPs = pathogen associated molecular patterns; DAMPs = damage (or danger)-associated molecular patterns; NK = natural killer cells]. Note: representative non-linear timeline scale.

In contrast to the 3–4 billion years of immune system evolution, for only a mere 60 years or so, humans have more intensively investigated the immune system and its intricate interplay between non-specific (innate) and more specific (adaptive) immune mechanisms for fundamental evolutionary and developmental advantage ( Figure 1). Often viewed as separate arms of the immune response, it is clear that they are rarely mutually exclusive or separate. The ‘artificial’ division arose for experimental explanatory research reasons, rather than physiological ones, but both are inextricably inseparable.

Genomic intrinsic mutational phenomena and exogenous infection of cells are significant forces capable of exerting phenotypic change to produce cell membrane 'aberration'. During cellular transformation to dysplasia, metaplasia and malignancy, cell membrane changes are detectable. Since gene mutations occur about 1 in every 10 ^6^ cell divisions, the risk of cellular aberration is high in rapidly dividing tissues, with some leading to malignant transformation. The immune system is the only system capable of high-level detection and action, and must therefore detect aberrant cells early and remove them exceedingly effectively and efficiently, otherwise, the rates of cancers would exceed that observed clinically. About 10 ^6^ cells form a 5mm diameter tumour mass from some 30 divisions (assuming a regular process applies).

Progressive exposure to environmental antigens in-utero, during infancy and subsequently, ultimately leads to acquisition of a very individual repertoire of antigens for which immunological tolerance and memory are established, thus shaping the immune system over time with the maturity of the individual enhancing adaptive immunity and survival. Recent data indicate that early antigenic exposure, including through the microbiome, transplacentally and during infancy is related to antigen tolerance and that lack of exposure is associated with higher allergic sensitisation and atopic reactions. There is also evidence that the level of antigen exposure (dose) is important in induction of T-cell responses and tolerant states to antigens ^46, 47^ and that progressive sensitisation with small repetitive doses of antigen can induce (variable) tolerance to serious peanut and bee-sting allergy ^48– 52^, with some relevance to the breaking of immune tolerance to cancer ^1, 53, 54^.

The link between the extent of environmental exposure to antigens shaping and developing the immune system and the incidence of disorders such as chronic infections, allergy and cancer has been extensively discussed ^49– 54^ and remain topics of considerable on-going interest.

## Fundamental Reactivity to Aberrant Antigens

Aberrations, arising from multiple events such as infection of cells, cellular injury, trauma, ageing or from genetic mutation, are reflected by cell surface expression of aberrant proteins, lipids (especially glycolipids) and carbohydrates. Detection of aberration through both non-specific and specific adaptive mechanisms is essential for destruction and removal of abnormal cells to restore tissue integrity. Membrane profile alterations from normal to dysplastic and malignant transformation are evident using magnetic resonance spectroscopy ^55– 57^. The immune system is carefully tuned to detect relatively subtle changes in proteins through the standard HLA systems via Class I and II molecules, and the far less explored CD1 system for the detection of lipid, glycolipid and carbohydrate molecules ^58^. In addition, the Fc receptor mechanism of the non-specific arm of the immune system detects foreign and altered cells. Activation of granulocytes, macrophages, B-cells and T-cells pushes the immune system in one direction or in the other, producing either overall responsiveness/activation, or inhibition/tolerance. Increasingly, it is being appreciated that almost all levels of the immune system can either respond or inhibit, providing a certain redundancy at multiple levels of control. Therefore, infected, damaged or malignant cells can be either actively eliminated or tolerated. Clinically, this is precisely what is observed, in a variety of infections and malignancies. Indeed, chronic inflammatory states have emerged as the predominant illnesses affecting many individuals, including persistent infections, autoimmunity and malignancy. Diseases such as cancer, cardiovascular disease and diabetes are now appreciated as chronic persistent inflammatory states, capable of modulation by factors such as anti-inflammatory medication and immune modulation.

## Evolutionary Roots of Cancer

In recent years there has been increasing interest in cancer as a biological phenomenon found across almost all multicellular species ^59^ suggesting deep evolutionary roots dating back to the dawn of multicellularity. The transition from unicellularity to multicellularity occurred several times starting over one billion years ago, although evolutionarily modern multicellular organisms with a large variety of cell and tissue types did not emerge until the Ediacara and Cambrian eras <600 million years ago. In 1929, Boveri ^60^ proposed that cancer represents a type of atavism or reversion to a more primitive ancestral phenotype. This general idea has now been widely confirmed using phylostratigraphy to study the evolutionary ages of cancer genes. It is generally recognized that the genes responsible for cellular cooperation in multicellular organisms (e.g. signalling, adhesion, angiogenesis, migration) are precisely those genes that are corrupted in cancer and lead to loss of regulatory function ^61– 63^.

In a series of papers ^64– 68^, Boveri’s idea of cancer as a type of atavism has been developed into a detailed theory of cancer onset and progression, which makes many quantitative predictions testable using phylostratigraphy.

It has long been recognized that there is a close link between cancer and early-stage embryogenesis ^69– 71^ which makes sense in the context of the atavism theory given von Baer’s 1828 so-called 4th law of embryology: “The embryo of a higher animal form never resembles the adult of another animal form, such as one less evolved, but only its embryo.” Although ‘law’ is an overstatement, Haekel’s much-criticised 1866 aphorism ‘ontogeny recapitulates phylogeny’ is nevertheless still a useful guide ^72^, and supported by evidence that complex interactions between genes, cells and developmental processes peak during mid-embryogenesis when the basic body plan of the organism is being laid down ^73^. The atavism theory predicts that as cancer progresses, cells de-differentiate towards ‘stemness’ and in a general sense resemble more closely the cells of both early stage embryogenesis and the unicellular world.

Phylostratigraphic analyses relevant to cancer began with the pioneering work of Domazet-Lošo & Tautz ^74^. They assigned ages to ‘cancer-associated genes’ from several cancer gene compilations and found two peaks where cancer genes are over-represented compared to the age distribution of all human genes. One was in the pre-eukaryote unicellular era. The other correlated with the origin of multicellularity and metazoa. The most important finding was the under-representation of cancer-associated genes younger than about 400 million years, confirming the basic notion that the evolutionary roots of cancer are very ancient.

Chen *et al*. set out to test the atavism model by examining xenograft human breast tumours in mice and characterized the complete evolutionary history of a tumour ^75^. The expression profiles were found to evolve towards that of embryonic stem cells. The most highly mutated and consistently downregulated genes in the metastatic samples were enriched in functions related to multicellularity. In another analysis, the phylogenetic tree of Tyrosine Kinases (TKs), which compose a major portion of oncogenes, demonstrated “a general trend of atavism in tumourigenesis” ^76^. Studies by Wu *et al*. of multiple myeloma evolving in a microfabricated ecosystem also supported the general thesis that “cancer represents a reversion back to ancient forms of life” ^77^.

An Australian group applied phylostratigraphy to RNA transcript sequencing data from The Cancer Genome Atlas for seven solid cancers, using 16 age categories ^78^. Consistent with Domazet-Lošo & Tautz ^74^, they found significant patterns in the relationship between gene age and expression levels in cancer. Genes of unicellular evolutionary origin are over-expressed in human cancers, whereas genes appearing at multicellular stages are down-regulated. The over-expression of unicellular genes was associated with major dysregulation of the control structures imposed on unicellular processes during the evolution of multicellularity. Significantly, Trigos *et al*. found that the atavistic signature was not a simple re-primitivisation to unicellularity. Rather it is a rewiring of the coupling between the gene networks that control unicellular processes from those that control multicellular processes ^79^. Their results thus provide evidence that cancer is a nuanced reorganization of the relationship between the unicellular and multicellular domains. A key prediction of this theory is that the reversionary sequence is systematic and should display regularities across species and across cancer types.

Zhou *et al*. ^80^ examined transcriptomes to compare differential gene expression between normal and cancer cells, and between embryonic and mature epithelial cells. Starting with 11 phylostrata, they made 5 age bins: LUCA, Eukaryota, Metazoa, Vertebrata, Primata, and then reduced these to two bins: pre-metazoan and post-metazoan. They found that compared to normal cells, cancer cells were enhanced in pre-metazoan and depleted in post-metazoan gene expression. Compared to embryonic cells, differentiated epithelial cells were enriched in post-metazoan expression. Their summary: “These findings support the atavism theory that cancer cells manifest the reactivation of an ancient ancestral state featuring unicellular modalities.”

Using phylostratigraphy, Cisneros *et al*. ^81^ found that recessive cancer genes older than 900 million years are homologs of the ancient genes in bacteria that turn up mutation rates when the cells are stressed–suggesting that the well-known genomic instability of cancer can be interpreted as a reversion to an ancient prokaryotic stress response, a phenomenon recently confirmed by the analysis of Cipponi *et al.* ^82^ showing mTOR (mammalian target of rapamycin) signaling orchestrates stress-induced mutagenesis, facilitating adaptive evolution in cancer.

The retention over evolutionary time scales of a DNA repair mechanism that *increases* the mutation rate in response to cellular stress may seem counter-intuitive. One reason is that the generation of somatic genome diversity is critical to the functioning of the immune system via somatic hypermutation of V regions to generate antibody diversity ^83^. Somatic genome diversity is also implicated in the implementation of polyploidy in megakaryocyte development ^84^, and the appearance of tetraploidy in normally functioning liver and cardiac tissues ^85^.

Cancer is conveniently characterized as displaying a set of distinctive hallmarks ^86^ which represent both gain and loss of function. Significantly, cancer does not evolve the hallmark properties *ab initio*; neoplasms merely appropriate pre-existing modalities latent in the genome ^75, 87^, retained because they play critical roles in key processes such as genetic diversity, embryogenesis, tissue maintenance and wound healing ^71, 88^.

A central prediction of the atavism theory is that there should be definite patterns in the direction, order and timing of both the loss and gain of function as cancer progresses, occurring via a sequence of increasingly malignant transformations ^89^. Cancer progression should roughly correlate inversely with the chronological sequence in which the relevant cancer genes evolved phylogenetically. By contrast, Hanahan & Weinberg (2015) remark: “The order in which these hallmark capabilities are acquired…appears to vary across the spectrum of human cancers.” Thus, the existence of non-randomness in hallmark acquisition provides a useful test to discriminate between the atavism theory and the prevailing somatic mutation theory. Evidence for the non-random nature of hallmark acquisition has been identified by Lineweaver and Chopra ^90^.

A well-known hallmark of cancer is its ability to evade or block the adaptive immune system, a feature that forms the basis of immunotherapy. Given the relatively young evolutionary age of adaptive immunity (< 500 million years), the atavism theory predicts that adaptive immunity should be lost soon after the onset of tumourigenesis ^65^. Another familiar hallmark is the default in cancer metabolism to anaerobic glycolysis even in the presence of normal oxygen tension, a phenomenon known as the Warburg effect ^91^. Glycolytic metabolism is well adapted to hypoxic environments of the sort encountered in tumours, but was also the normal circumstance for life before the great oxygenation event about 800 million years ago. By upregulating the Warburg effect, cancer can be regarded as reverting to the ancient hypoxic roots of early multicellular life. Indeed, it has been argued by Vincent ^65^ that cancer engineers its tissue microenvironment to recreate congenial atavistic niches in which neoplasms can flourish in competition with healthy cells.

The atavism theory has important implications for therapy ^65^. The history of the interaction of bacteria, viruses and cancer is a very long and somewhat confused one, since William Coley obtained some notable clinical results over a century ago ^92^. Some infections will boost the immune system and bring additional pressure on cancer cells, but some agents will directly infect the cancer cells preferentially in their immunosuppressed niches, for example oncolytic viruses. A variety of new approaches ^1, 93– 99^ to immunotherapy exploits these features. The atavism theory predicts that advanced cancer will be particularly vulnerable to certain infectious agents, and specific treatment regimes have been advocated to take advantage of that aspect ^65^.

We also note that, taking into account the discovery of stress-induced mutagenesis – a very ancient stress response found in bacteria and pre-dating multicellularity – the practice of maximum tolerable dose for chemotherapy is likely to be counterproductive. It risks provoking an elevation of the mutation rate (another hallmark of cancer), thus facilitating the ability of the neoplasm to evolve drug resistance. Instead, a more measured and nuanced treatment regime is likely to be more efficacious.

## Contemporary Cancer Evolution

Not only is there an evolutionary past of considerable longevity, but the tumour deposit itself has frequently been demonstrated to have wide genetic heterogeneity arising from the initially clonal multi-cellular growth, and moreover different metastases show considerable heterogeneity between them. It is therefore evident that the ‘cancer’ is not truly clonal, but represents a mix of cells resulting from genetic instability with different genetic profiles, which the immune system must recognise in order to react to remove the constituent cancer cells within the tumour mass(es) ^100– 104^. Cancer phenotype has been proposed as an evolutionary interaction between normal and malignant cells ^105^, and furthermore, recent evidence indicates that the hierarchical structures in normal host tissues are structurally mirrored in a very similar way by tumour tissues ^106^. Additionally, mathematical models have been developed to investigate how de-differentiation of tumour cells appears to be an adaptive mechanism actively selected-for to permit invasion into cellular hierarchies ^106^.

## Homeostatic Regulation of Immune Reactivity and Cancer

The relapsing and remitting behaviour of many chronic inflammatory states, such as arthritis, inflammatory bowel diseases, multiple sclerosis, and thyroiditis is well recognised. Diabetes, cardiovascular diseases and cancers of all types are now being considered similarly. The fluctuating, oscillating nature of these diseases has largely confounded our understanding to date and remained frustratingly unexplained, but is indicative that the immune system must be repeatedly transitioning between stimulation/activation and suppression/tolerance phases repeatedly to produce the observed clinical picture. Moreover, oscillatory behaviour is highly characteristic of any homeostatic biological system under negative feedback control. This cyclical dynamic is a physical expression of physiological control to maintain relative constancy of the *milieu intérieur,* as recognised by Claude Bernard around 1867, and later by Walter Cannon. Physiological constancy, or homeostatic control, of the body's immune status requires proportioned synchrony between effector stimulation *and* regulatory functions to be operational. Many cyclical examples, such as the diurnal temperature cycles, peri-monthly menstrual cycles, and 24-hour cortisol cycles have been elucidated by close serial monitoring. The fluctuating pattern in these situations defines the “set-point” which that specific physiological parameter is maintained at to define the mean and standard deviation of that variable.

The association between cancer and the host immune response has been recognised for over a century ^107– 112^. In animals, North *et al.*’s group ^113– 121^ and more recently Klatzmann *et al.*’s group ^122^, demonstrated that the timing of delivery of cytotoxic agents after tumour transplantation was crucial in determining whether tumour regression occurred or not. Early clinical observations of inflammation and cancer regression were made by those treating cancer ^107– 110^, particularly the development of infection/fever after surgery. Chronic inflammation has long been associated with cancer development, for example chronic ulceration and Marjolin's squamous cell cancer of the skin.

The immune system has innate and adaptive arms. Chemicals are released into the serum during inflammatory responses and can be used as clinical and laboratory markers of the presence and strength of inflammation. These can be used to infer the rate of increase or decrease of inflammation that is occurring at the time and are dependent on the half-lives of the relevant marker(s), and often other factors such as organ function. Acute phase markers include C-Reactive Protein (CRP), serum amyloid A, complement factors, ferritin, ceruloplasmin, erythrocyte sedimentation rate, white blood cell count, haptoglobin, immunoglobulin and a range of cytokines released into the serum and/or tissue. Many of these tests are routinely used clinically for measurement of inflammation and for monitoring of treatment and disease progression.

One of the easiest to measure, is the relatively reliable and clinically widely useful acute phase marker CRP, a non-specific functional analogue of immunoglobulin that binds to self/non-self cellular breakdown products of inflammation to initiate the adaptive immune responses ^123, 124^. T & B cells respond to cellular changes due to infection, damage or mutagenesis. To fine-tune and limit these responses, the ensuing immune response is down-regulated paradoxically by the same cytokines and receptors that initiated it, **but** on functionally different cell types. Regulatory T-cells play a major role in this homeostatic attenuation and experimental and clinical evidence has shown that when these cells are either removed or blocked, cancer can completely regress, while autoimmune conditions may develop or worsen ^125– 130^.

In recent years, it has become clear that the immune system recognises and processes both self- and non-self antigens to either respond or tolerate the antigen, but that homeostatic balance usually prevails.

**Figure 2.**
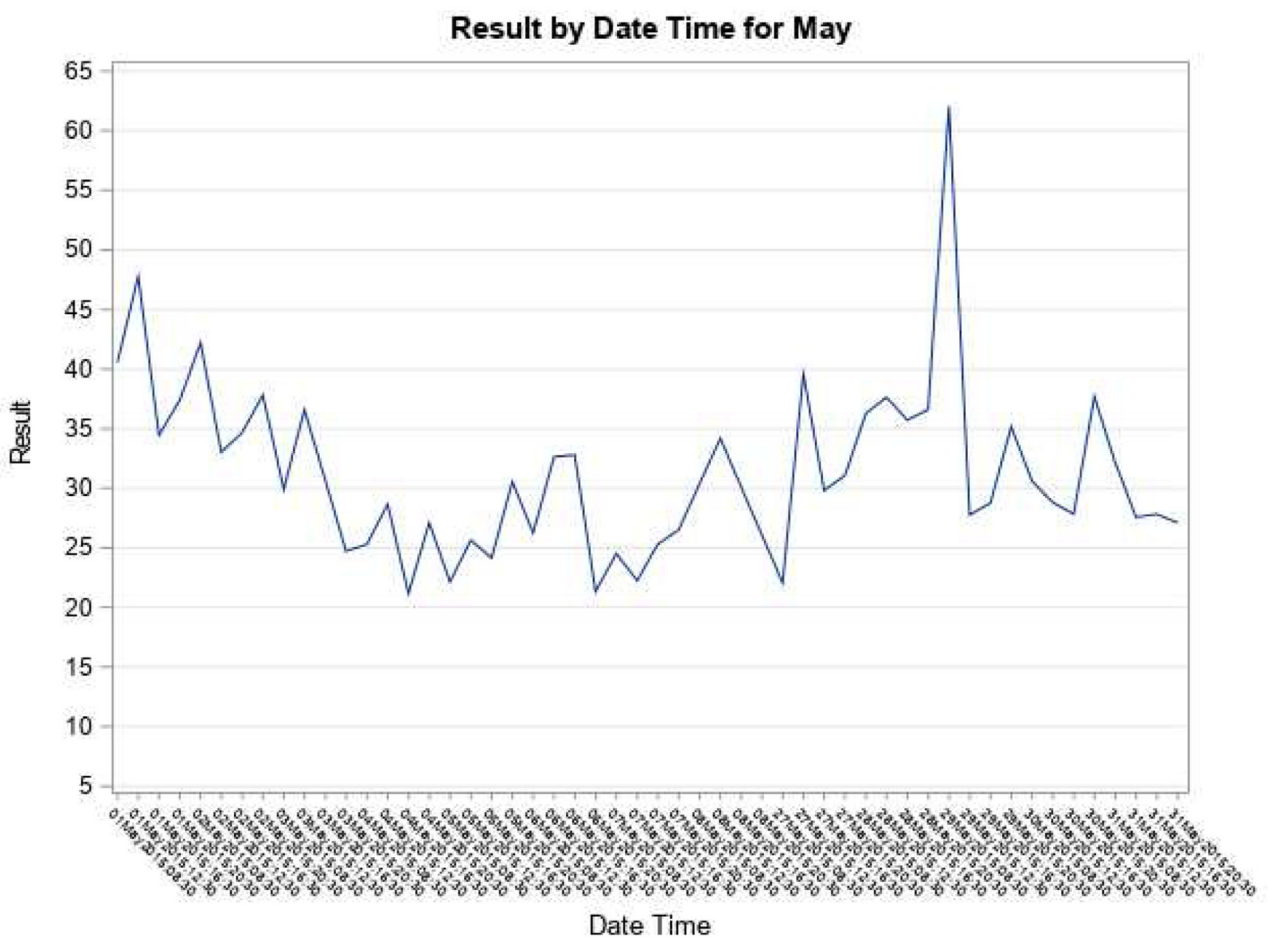
C-Reactive Protein (CRP) Level Fluctuation. An example of CRP oscillatory behaviour in a patient with cancer - an example of the immune system fluctuating with a varying inflammatory response to an antigen stimulus. Several CRP levels (results) were taken each day. Antigen is capable of driving activation or inhibition depending on the strength and timing of the signal input and on operational feedback facilitatory and inhibitory loops, alternately driving both responsiveness and tolerance to create net homeostasis around a set-point mean. Multiple cytokines (eg. IL-2, IL-7, IL-10; IL-12; IL-17; IL-21; IFN-γ) and other molecules (eg. anti-PD-1, anti-CTLA4, antigen) have the capability of either activating or inhibiting immune responses, so are bi-functional.

Immune responses can therefore be thought of as an oscillating or dual-phase “bi-stable" system existing in either of two principal states (responsiveness or tolerance). Antigen is the prime mover for either of these two states, and cytokines, most notably interleukin-2 (IL2) as an example (but also including IL-7, IL-10; IL-12; IL-15; IL-17; IL-21; IFN-γ) and other molecules (eg. anti-PD-1, anti-CTLA4), provide the temporal feedback loop to govern the immune response direction. If antigen is continuously supplied to such a system (due to tumour cell growth/ turnover) logic and physiology dictate that this response must oscillate ^123, 124, 131– 134^. Oscillatory systems are characteristic of any homeostatic system with feedback loop(s) ( Figure 2).

Not only is the immune response governed by ‘when’ the cytokine or antigen signals are delivered, but also by the strength (how much) of the signal that is delivered. Small quantities of cytokine, IL-2 for example, induces a different effect to a larger quantity of the same cytokine – a point exploited to some degree in IL-2 therapies ^135– 138^.

## Anti-cancer Agents and Immune Responses

Cytotoxic agents inhibit cell division to therapeutically damage and kill tumour cells. However, cancer cells divide asynchronously. About 20–30% of malignant cells within many solid cancers are dividing at any one time-point (greater rates of division occur in some cancers such as childhood leukaemia, choriocarcinoma and testicular carcinoma). Regimens have evolved often with weekly dosing of sequential 'lines' (1st, 2nd, 3rd etc) of therapy or in combinations. Repetitive dosing of agents inducing multiple cycles of cell damage and antigen release (vaccination events) from the tumour is emerging as highly significant ^1, 54, 123, 131^.

Cells of the immune system rapidly divide, but they usually divide synchronously and the arms of the immune response proliferate alternately (effector then regulatory) at different times sequentially to initiate then reduce an immune response over time ^123, 124, 131– 134^.

Cytotoxic agents, as well as desirably ablating actively dividing cancer cells, unless applied discriminately, are capable of ablating different groups of proliferating immunological cells, including proliferating effector T-cells.

It is now clear that the immune system is not ignorant to the presence of tumours, that it does recognise tumour antigens, and that the normal homeostatic regulatory mechanisms suppress active anti-tumour immune responses, and are at the seat of the problem. This explains why immuno-modulatory agents, such as IL2, and CTLA4, PD1/L1 monoclonal antibodies can deliver random dramatic complete responses in a limited percentage of late-stage cancer patients by releasing the pre-existing homeostatic suppression/tolerance ^1, 125– 130^. All of these immunotherapeutic agents can induce immune activation, but can also induce immune tolerance. The lack of efficacy of these agents in most patients is explained by induction of tolerance with some doses via regulatory T-cells while activating with other doses, the net balance of which might determine overall clinical outcome.

The application of immune therapies or cytotoxic chemotherapies to the oscillating and fluctuating immune system occurring in the individual patient presents a significant problem if the action of the specific agent depends on precisely when the agent is administered with reference to the phase of activation or inhibition of the immune system at the time. Recent work has been unable to accurately define the periodicity of the immune oscillation in cancer patients despite initial work using less robust mathematics appearing to show this ^139– 142^. Advanced mathematics and periodicity models have been incapable of verifying regular, consistent, clear, regular immune cycles, principally due to infrequent inflammatory marker serial sampling and confounding noise-to-signal issues, but machine (deep) learning may offer alternative methods. On-going work into the oscillatory behaviour of the immune system, especially in advanced cancer patients, may reasonably offer useful opportunity to understand and to forecast or predict treatment intervention more accurately than exists at present ^143^.

## Improving results of natural selection

Tissues in the body are usually maintained with a very uniform cell composition. Most aberrant cells appearing in the human body as a result of somatic mutations are detected and disposed of by the immune system. Some are not and can produce pathology, with the majority of clinical cases of cancer occurring in older patients. This is explicable by the fact that natural selection operates principally by differential reproduction, consequently it is unable to operate for selection of biological characteristics in non-reproductive (older) individuals. Thus, over the generations immune responses to malignant cells appearing in young people became adjusted by natural selection and, statistically speaking, operate efficiently, while such responses in older age were not “reachable” by natural selection for genetic adjustment. This principle is not only applicable to specific immune responses, but encompasses the entire regulation of homeostatic balance of an organism. In practical terms, clinical intervention should imitate adaptation by selection of immunological processes occurring in younger organisms, to support, adjust and enhance natural operation of immune systems of older patients. Since the onset of significant clinical interventions about a century ago, death has been reduced such that the operational pressures of natural selection have been relaxed and, as one of the consequences, the incidence of many cancers has increased ^19^, while concurrently improved treatments have also extended the lives of many patients.

## Concluding Remarks and Implications

Although knowledge has deeply developed concerning the immune system and cancer immunology, our contemporary understanding needs to be placed in evolutionary perspective. Our immune systems are the adaptive result of the necessity for defence against persistent selective pressures from environmental microbial pathogens, injury and repair. Over the millennia, the immune system and other body cells have undergone a continuous adaptive symbiotic process of synchronous, coordinated, cooperative, progressive immunological and somatic evolutionary change to provide what we observe today. Gradual evolution of innate and adaptive immunity against infected and aberrant cells now explains many of the observations regarding cancer immunity and clinical responses. It is gradually being appreciated that normal immune regulatory mechanisms are holding back an already primed immune response from selectively killing cancer cells in most, if not all cases, where clinical cancer is present. With an appreciation that immuno-modulation of pre-existing endogenous immune responses appears to occur with most cancer therapies, there is the serious prospect that serial immune monitoring might define optimal time-points for targeted administration of therapies to engineer effective complete clinical responses in a much more predictable, reliable and durable manner in the future ^1^. Effective treatments may indeed hinge on deep evolutionary immune and cellular mechanisms which likely underpin successful clinical responses. If achievable, increased long-term survival from advanced cancer, with reduced toxicity, might become a reality by harnessing the immuno-modulatory capacity of many currently existing therapeutic agents. The cost savings would likely prove truly enormous ^144^.

## Authors’ information

The authorship of Version 1 represented a unique collaboration between diverse disciplines with contributors having backgrounds and qualifications in cancer surgery, immunology, immunotherapy (BJC), laboratory science (MLA), evolutionary biology, anatomy, anthropology (MH), and the physical sciences, cancer biology (PCWD). BJC & MH updated and rewrote Version 2 and responded to the reviewers comments; we thank PCWD who did not want to be listed as an author on Version 2, but contributed to the section on cancer cell evolution. As such, this work aims to approach the problem of cancer development and immune system recognition/responses uniquely from a scientific evolutionary perspective to explain many of the clinical observations that have been already made to date. Emanating from this understanding, new approaches and therapies might then be fruitfully generated for science and clinical medicine.

## List of abbreviations

IL2         interleukin-2

CTLA4  cytotoxic T-lymphocyte associated protein 4

PD-1      programmed cell death protein 1

PD-L1    programmed death-ligand 1

HLA       human leukocyte antigen

CD1       cluster of differentiation 1

DNA       deoxyribonucleic acid

EMT       epithelial-mesenchymal transition

MET       mesenchymal–epithelial transition

mTOR    mammalian target of rapamycin

PS-OC    Physical Science-Oncology Centers

HIV        human immunodeficiency virus

AIDS     acquired immunodeficiency syndrome

HREV     human endogenous retroviruses

Ma          mega/million years

Ga          giga/billion years

## Author contributions

BJC devised and wrote the main text; MLA contributed to the initial (Version 1) discussion on HIV/retroviruses and hyper-variable T-cell receptors (now heavily amended); MH contributed to evolutionary discussions; PCWD contributed to cancer cell evolution. All authors prepared, read and approved the manuscript to reach the final (Version 1) content; BJC, MH and PCWD reviewed, edited and approved the manuscript revision (Version 2); *MLA was involved in a minor way with the first version with sections now extensively amended, and not with the latest revised versions (Versions 2 & 3).

## Author information

CSO Biotempus Ltd, the institutional affiliation for MA, has gone into liquidation since version 1 of this article was published.
