# Supplementary material for: The Immune System and Responses to Cancer: Coordinated Evolution
Source: F1000Res. 2021 Jan 27;4:552. Originally published 2015 Aug 12. [Version 3] doi: 10.12688/f1000research.6718.3 (PMC7735224; doi:10.12688/f1000research.6718.3)
Supplement: Peer review history

# Peer review: Reviewer response for version 2

**DOI:** 10.5256/f1000research.29628.r74419

**Published:** 2020-12-11

**Authors:** Angus Dalgleish

**Affiliations:** 1 Department of Oncology, Institute for Infection and Immunity, St George's University of London, London, UK

This will now provide a valuable source for investigating the evolutioanry link between the immune system and cancer.

 The section on time line evolution reporting in "Darwins" and millions of years etc, could be made more impactful by using a figure to capture the scale and of genomic, immune response in living organsisms.

Reviewer Expertise:

NA

I confirm that I have read this submission and believe that I have an appropriate level of expertise to confirm that it is of an acceptable scientific standard.

# Peer review: Reviewer response for version 2

**DOI:** 10.5256/f1000research.29628.r74418

**Published:** 2020-11-12

**Authors:** Jonathan M. Austyn

**Affiliations:** 1 Nuffield Department of Surgical Sciences, John Radcliffe Hospital, University of Oxford, Oxford, UK

Some comments on immunological aspects (only):

1. Recommend use of the generic term 'patter-recognition receptors, PRRs' instead of the more-specific term 'toll-like receptors, TLRs, which are a subset of PRRs.
2. Helpful to distinguish between somatic recombination - for generation of BCR and TCR, and analogous process for antibody class switching - and somatic hypermutation which should be restricted to antibody V regions, at least in humans. (There is evidence for somatic hypermutation of TCRs in shark alpha chains).

Reviewer Expertise:

NA

I confirm that I have read this submission and believe that I have an appropriate level of expertise to confirm that it is of an acceptable scientific standard.

# Peer review: Reviewer response for version 1

**DOI:** 10.5256/f1000research.7216.r10255

**Published:** 2015-09-10

**Authors:** Angus Dalgleish

**Affiliations:** 1 Department of Oncology, Institute for Infection and Immunity, St George's University of London, London, UK

I was asked to review this article, with full access to previous reviewer's report, Jonathan M Austyn, from the John Radcliffe Hospital. I agree that the article contains some very interesting ideas and concepts, which will be very stimulatory to a wide readership, particularly those studying cancer and practicing cancer (oncologists) who are slowly becoming aware that the immune system is important in the control of cancer and hence the development and evolution.

The review touches on some very broad and very interesting concepts, particularly with regards to evolution over time and the evolutionary difference between innate and adaptive immune systems and he has some of the concepts that are relevant to the hypothesis but perhaps not been elucidated that clearly, especially with regards to the concept of atavism and this has been pointed out by Jonathan Austyn with regards subtle differences in somatic recombination and hypermutation and mutation.

I also agree with rather more reaching, throwaway statements, such as 'cancer occurs in all humans and animals, thus remaining a mystery when several reasons have already been explored, such as random mutations and the concept of escaping tumour surveillance.cancer

Whereas references made to many infectious agents and the fact that they have ended up making up the vast majority of the intron genome, which has been shown to be due to the incorporation of many virus and bacterial sequences.  The hypothesis depends very much on the shaping of these agents and the immune system but does not deal adequately with how the immune is shaped by exposure to infectious agents during infant and childhood development. 

Much of the references of this article is in large chunks, whereas the authors' papers are listed in large batches, for example; the work by North and colleagues are all listed together and referenced together.

I feel that in addressing this, that they have not acknowledged the work of others who have spent a long time working out the affects of basic infectious diseases on the immune system and how this translates to chronic infectious diseases and one author in particular stands out for his work on this and that is Graham Rook, he has published much on this about the exposure of pathogens and the dirt theory, how it impacts on chronic diseases in later life in the western world and, indeed, a review by him and this reviewer ( Rook and Dalgleish, 2011) published in Immunological reviews in 2011 goes into great detail with regards to its impact on cancer.

Similarly, the impact of both infectious and non-infectious chronic activation/inflammation, which is relevant to many of the issues raised in this article, have not been addressed sufficiently, especially as it is so relevant to many of the speculations discussed.  Again, there are a large number of authors and contributors who have gone from broad brush theories of the association of chronic inflammation and cancer, to those who have gone into great detail pointing out how it impacts on the molecular level of mutations in suppressor genes, such as P53, all the way through to immunological hypoxic pathways, etc., and I do not recognise this from the reference list which, as mentioned, is very block buster in its approach with an author's several contributions all being listed together throughout.

I also feel that, with regards to the homeostatic component focusing purely in Interleukin-2, that although it is true that it is one of several cytokines which contribute to activation and tolerance that a little bit more background should be given.

In conclusion, I think this is a very valuable piece of work of great interest to the rapidly proliferating and emerging population of cancer specialists who are slowly becoming aware that the immune system is extremely important in the management of this disease, a concept that has been ignored for the last few decades and foreign to most oncologist practising today. However, to make it more impactful and a 'must read' article it does require considerable focusing on the aspects raised by the referees and tighter structure, as it does seem to read rather like a speculative lecture in its current format.

With regards to referencing, there is a referral to Klazmann *et al.* and there is no Klazmann *et al.* in the references and this is a further example that the references need to be very carefully looked into, although a paper where he is the senior author is listed, I do not think that it is appropriate therefore to refer to it in the text as Klazmann *et al*.

Reviewer Expertise:

NA

I confirm that I have read this submission and believe that I have an appropriate level of expertise to confirm that it is of an acceptable scientific standard, however I have significant reservations, as outlined above.

# Peer review: Reviewer response for version 1

**DOI:** 10.5256/f1000research.7216.r9959

**Published:** 2015-08-13

**Authors:** Jonathan M. Austyn

**Affiliations:** 1 Nuffield Department of Surgical Sciences, John Radcliffe Hospital, University of Oxford, Oxford, UK

This article contains some very interesting ideas and concepts. However, to this reviewer (and with the greatest respect to the authors), it tends to read perhaps as ‘stream of consciousness’ writing rather than a considered scientific article. The authors show abundant enthusiasm in their writing, but this often appears to mask real scientific rigour. It is very difficult to identify a key hypothesis (or hypotheses), the structure of the sections seems to lack definition or focus, and it is sometimes almost impossible to understand what the main conclusions are from each. Furthermore, in a number of places, there seems to be a significant lack of scientific accuracy. For example, regarding some statements regarding the immune system, the authors do not clearly discriminate between somatic recombination (which applies to both T cell receptors and B cell receptors), somatic hypermutation (which applies only to the latter), hypervariable regions (which are present in both) and mutation ( *per se*). As another example, in their discussion of atavism theory, the authors use the term ‘tumour immunosuppression’ but appear to apply this to the tumour rather than the host, where it really belongs. Even the authors’ brief review of the evolution of the immune system seems to lack sufficient focus going from species to species during evolutionary time. There are also some rather vexing ‘throwaway statements’. As just one example, regarding cancer, the authors state “The reason why cancer occurs at all in humans and animals thus remains a mystery”. While it is certainly true that much remains unknown, the authors might usefully consider further Burnet’s concept of tumour surveillance, particularly in its more recent form comprising immunoediting, equilibrium and escape phases. It might also be valuable to reconsider, in relation to the immune system’s apparent capacity to eliminate tumours whether or not the immune system actually helps to eliminate the infectious agents (e.g. viruses) that can cause tumorigenesis rather than actual or potentially malignant cells (and, on that point, it is perhaps a little surprising that there is no mention of generic ‘DAMPs’ and ‘PAMPs’). A further criticism is that the choice of immunological mechanisms under discussion sometimes feels rather random – why, for example, do the authors specifically focus on Fc receptors rather than complement receptors, or C-reactive protein rather than the many other molecules that play similar or related roles? Finally, regarding the single diagram that is presented, it is completely unclear why the authors have chosen to illustrate an ‘IL-2 [sic] feedback loop’; without any justification this seems to be over-simplistic in the extreme. Nevertheless, to return to the initial point: this article does appear to contain some very interesting material that would potentially be of value to readers of the journal. To do these the greatest justice, however, really does seem to require a very careful, focussed, and considered rewriting of the present text into a completely revised and possibly restructured article.

Reviewer Expertise:

NA

I confirm that I have read this submission and believe that I have an appropriate level of expertise to confirm that it is of an acceptable scientific standard, however I have significant reservations, as outlined above.
